# Integrated Biomarker–Volumetric Profiling Defines Neurodegenerative Subtypes and Predicts Neuroaxonal Injury in Multiple Sclerosis Based on Bayesian and Machine Learning Analyses

**DOI:** 10.3390/biomedicines14010042

**Published:** 2025-12-24

**Authors:** Alin Ciubotaru, Roxana Covali, Cristina Grosu, Daniel Alexa, Laura Riscanu, Bîlcu Robert-Valentin, Radu Popa, Gabriela Dumachita Sargu, Cristina Popa, Cristiana Filip, Laura-Elena Cucu, Albert Vamanu, Victor Constantinescu, Emilian Bogdan Ignat

**Affiliations:** 1 Grigore T. Popa University of Medicine and Pharmacy, 700115 Iasi, Romania; alinciubotaru94@yahoo.com (A.C.); daniel.alexa@umfiasi.ro (D.A.); victor.constantinescu@umfiasi.ro (V.C.); emilian.ignat@umfiasi.ro (E.B.I.); 2 Department of Radiology, Biomedical Engineering Faculty, Grigore T. Popa University of Medicine and Pharmacy, 700115 Iasi, Romania; ana.covali@umfiasi.ro; 3 Department of Morphofunctional Sciences, Grigore T. Popa University of Medicine and Pharmacy, 700115 Iasi, Romania; laura.knieling@umfiasi.ro; 4 Doctoral School, Grigore T. Popa University of Medicine and Pharmacy, 700115 Iasi, Romania; robert-valentin.bilcu@d.umfiasi.ro (B.R.-V.); dudau.laura-elena@d.umfiasi.ro (L.-E.C.); 5 Department of Vascular Surgery, Grigore T. Popa University of Medicine and Pharmacy, 700115 Iasi, Romania; radu.popa@umfiasi.ro; 6 Department of Obstetrics and Gynecology, Elena Doamna Obstetrics and Gynecology University Hospital, Grigore T. Popa University of Medicine and Pharmacy, 700115 Iasi, Romania; gabriela.dumachita@umfiasi.ro; 7 Faculty of Dental Medicine, Grigore T. Popa University of Medicine and Pharmacy, 700115 Iasi, Romania; cristina.popa@umfiasi.ro; 8 Department of Biochemistry, Grigore T. Popa University of Medicine and Pharmacy, 700115 Iasi, Romania; cristiana.filip@umfiasi.ro; 9 Basic and Clinical Neuroscience Department, Institute of Psychiatry, Psychology and Neuroscience, King’s College London, London SE5 8AF, UK; albert.vamanu@kcl.ac.uk

**Keywords:** multiple sclerosis, serum neurofilament light chain, brain volumetry, Bayesian analysis, machine learning, endophenotypes, neurodegeneration, predictive modelling

## Abstract

**Background**: The clinical–radiological paradox in multiple sclerosis (MS) underscores the need for biomarkers that better reflect neurodegenerative pathology. Serum neurofilament light chain (sNfL) is a dynamic marker of neuroaxonal injury, while brain volumetry provides structural assessment of disease impact. However, the precise link between sNfL and regional atrophy patterns, as well as their combined utility for patient stratification and prediction, remains underexplored. **Objective**: This study aimed to establish a multimodal biomarker framework by integrating sNfL with comprehensive volumetric MRI to define neurodegenerative endophenotypes and predict neuroaxonal injury using Bayesian inference and machine learning. **Methods**: In a cohort of 57 MS patients, sNfL levels were measured using single-molecule array (Simoa) technology. Brain volumes for 42 regions were quantified via automated deep learning segmentation (mdbrain software). We employed (1) Bayesian correlation to quantify evidence for sNfL–volumetric associations; (2) mediation analysis to test whether grey matter atrophy mediates the EDSS–sNfL (Expanded Disability Status Scale) relationship; (3) unsupervised K-means clustering to identify patient subtypes based on combined sNfL–volumetric profiles; and (4) supervised machine learning (Elastic Net and Random Forest regression) to predict sNfL from volumetric features. **Results**: Bayesian analysis revealed strong evidence linking sNfL to total grey matter volume (r = −0.449, BF_10_ = 0.022) and lateral ventricular volume (r = 0.349, BF_10_ = 0.285). Mediation confirmed that grey matter atrophy significantly mediates the relationship between EDSS and sNfL (indirect effect = 0.45, 95% CI [0.20, 0.75]). Unsupervised clustering identified three distinct endophenotypes: “High Neurodegeneration” (elevated sNfL, severe atrophy, high disability), “Moderate Injury,” and “Benign Volumetry” (low sNfL, preserved volumes, mild disability). Supervised models predicted sNfL with high accuracy (R^2^ = 0.65), identifying total grey matter volume, ventricular volume, and age as top predictors. **Conclusions**: This integrative multi-method analysis demonstrates that sNfL is robustly associated with global grey matter and ventricular volumes, and that these measures define clinically meaningful neurodegenerative subtypes in MS. Machine learning confirms that a concise set of volumetric features can effectively predict neuroaxonal injury. These findings advance a pathobiology-driven subtyping framework and provide a validated model for using routine MRI volumetry to assess neuroaxonal health, with implications for prognosis and personalised therapeutic strategies.

## 1. Introduction

Multiple sclerosis (MS) is a long-term inflammatory and neurodegenerative disorder of the central nervous system, characterised by complex interactions involving acute demyelination and ongoing axonal degeneration. While standard MRI assessments of T2 hyperintense lesions are essential for clinical observation, they are often regarded as not correlating well with the progression of clinical disability. This discrepancy is referred to as the clinical–radiological paradox [1]. As a result, there has been increased interest in identifying biomarkers that more accurately reflect the neurodegenerative aspects of the disease.

In this context, the light chain of serum neurofilament (sNfL) has become a prominent candidate. Neurofilaments serve as structural elements of the neuronal cytoskeleton, and their presence in cerebrospinal fluid and blood indicates neuroaxonal damage. Technological advances in ultrasensitive single-molecule array (Simoa) assays have facilitated the precise measurement of sNfL, confirming it as a sensitive, dynamic, and noninvasive biomarker of disease activity in MS [2,3]. Elevated sNfL concentrations are associated with acute relapses and radiographic evidence of disease activity and can predict future disease progression [4,5].

At the same time, brain volumetry has become an essential indicator of neurodegenerative aspects of MS. Continuous brain volume reduction, which occurs at a rate 3–5 times faster than in healthy groups, is a strong predictor of lasting physical and cognitive impairment [6,7]. More specifically, grey matter shrinkage is a stronger indicator of clinical dysfunction than white matter lesions or total brain atrophy [8,9]. Measuring brain volume in absolute values (cm^3^) and as age-, sex-, and intracranial volume-adjusted percentiles provides a structural reference point for validating fluid biomarkers.

The conceptual link between the fluctuating biochemical indicator of sNfL and the aggregate structural assessment of brain volume reduction is inherently compelling. Although initial research has established a negative cross-sectional relationship between sNfL and total brain volume, the exact nature of this link remains poorly defined [10,11]. Crucial questions remain: Which volumetric areas are most closely associated with increases in sNfL? Are there distinct subgroups of patients characterised by unique sNfL–volumetric profiles that could explain clinical variability?

Is it possible to go beyond simple correlation to create predictive models that assess neuroaxonal injury using a minimal collection of imaging features?

To answer these questions, it is necessary to go beyond traditional statistical techniques. Frequentist methods, centred on testing the significance of the null hypothesis, are limited in their ability to measure evidence for the null and are not ideal for large datasets. Therefore, this research utilises a multi-method analytical framework to provide a more detailed and impactful examination.

Therefore, this study uses a multi-methodological analytical framework to provide a more nuanced and robust investigation:

- Bayesian factor analysis is used to quantify the strength of evidence for correlations, overcoming the dichotomous “significant/insignificant” limitation of *p*-values and allowing for the interpretation of evidence in favour of alternative and null hypotheses [12].
- Unsupervised machine learning (cluster analysis) is applied to identify data-driven patient endophenotypes based on their combined sNfL and volumetric profiles. This method has proven successful in deconstructing MS heterogeneity into meaningful pathobiological subtypes [13].
- Supervised machine learning (regularised regression and Random Forests) is implemented to build predictive models, identify the most informative volumetric features for sNfL prediction, and capture potential nonlinear relationships, an approach increasingly recognised for its utility in MS neuroimaging [14,15,16].

We propose that this comprehensive, data-driven approach will demonstrate that sNfL levels are primarily predicted by a specific set of volumetric measures, particularly those indicating deep grey matter and cortical health, and will uncover unique groups of patients with varying risks of neuroaxonal injury or loss of function. These results aim to improve our understanding of the link between structure and function in MS and contribute to the creation of a multimodal biomarker framework for prognosis and personalised therapeutic approaches for people with MS.

The main objective of this research was to conduct an in-depth, multi-method approach to explore the link between sNfL, a “fluctuating marker” of neuroaxonal damage, and quantitative brain volumetry, a structural measure of disease impact in MS. We proposed to go beyond established correlational connections and to create predictive sensitive models and identify clinically meaningful patient subgroups, thereby addressing the essential requirement for a more detailed pathobiology which is focused on understanding MS diversity.

Measurement of relationship intensity and specificity is achieved by using Bayesian correlation analysis to accurately quantify the evidence of associations between sNfL and a comprehensive set of global and regional brain volumes, thereby identifying the most sensitive structural correlates of neuroaxonal injury that exceed the limits of conventional *p*-value that can be burdened by interference.

Clarifying the principal pathobiological pathways is achieved by exploring the mechanistic link between clinical impairment, brain shrinkage, and neuroaxonal injury by examining the hypothesis that grey matter volume influences the relationship between EDSS scores and sNfL concentrations.

Analysing disease diversity/heterogeneity is achieved using unsupervised machine learning (cluster analysis) to recognise data-driven patient “endophenotypes” derived from combined sNfL and volumetric profiles and to describe these subgroups in terms of clinical severity, cognitive ability function, and therapeutic approaches.

This research presents multiple new and significant components:

- Sophisticated statistical integration: This is one of the pioneering studies that combines Bayesian inference, mediation analysis, and unsupervised and supervised machine learning in a unified analytical framework to explore the connection between sNfL and cerebral volumetry. This diverse approach enables us to identify connections, quantify evidence, uncover underlying mechanisms, delineate subgroups, and simultaneously develop predictive models.
- We analysed the transition from association to prediction and classification. While prior research has shown cross-sectional correlations, our study focuses on predicting sNfL levels using volumetric data and on classifying patients into distinct pathobiological subtypes. This approach moves the research from simply identifying relationships to creating sensitive tools that could be clinically valuable for prognosis and patient stratification.
- Data-driven phenotyping through the integration of biomarkers and MRI imaging has led to the identification of subgroups such as “High Neurodegeneration” and “Benign Volumetry.” This approach relies on the seamless integration of fluid biomarkers and structural imaging, resulting in a more biologically relevant subtyping system than those that depend solely on clinical progression or standard MRI. This integration may offer a solution to the challenges posed by the clinical–radiological paradox.
- By employing regularised regression and ensemble methods on a multidimensional matrix of volumetric features, we have identified a concise set of key predictors, specifically global grey matter and ventricular volumes. This finding is crucial for the design of future studies, suggesting that a focused examination of these critical structures may account for a significant portion of the variation in neuroaxonal injury.

Elucidating the Reasons, Knowledge Deficiencies, Innovations, and Contributions

Although the importance of serum neurofilament light chain (sNfL) and brain volumetry in MS is well recognised, notable gaps remain. The exact connection between sNfL, a fluctuating indicator of neuroaxonal damage, and the composite structural metric of distinct regional atrophy patterns remains mechanistically unclear. Secondly, there is an absence of analytical models that combine these complementary methods to extract pathobiology-based disease subtypes and create predictive tools with real-world clinical applications. Although general cross-sectional correlations have been documented, a detailed analysis focusing on pinpointing the structural areas most responsive to sNfL changes and utilising them for patient categorisation and forecasting is still insufficiently investigated.

This research presents an important methodological advancement by integrating four complementary analytical methods—Bayesian inference, mediation analysis, and both unsupervised and supervised machine learning—into one cohesive framework. Transcending the common frequentist statistics and basic correlations found in earlier studies, this multimodal approach facilitates (a) thorough measurement of evidence supporting and opposing associations; (b) exploration of underlying mechanisms; (c) autonomous identification of patient subgroups; and (d) development of strong predictive models.

The main contributions of this study are four in total:

Identification of Primary Structural Correlates: It presents strong Bayesian evidence designating overall grey matter and lateral ventricular volumes as the principal structural correlates of sNfL, emphasising grey matter pathology as a primary factor in neuroaxonal damage.

Clarification of an Essential Mechanism: Utilising mediation analysis, it shows that grey matter deterioration notably mediates the connection between clinical disability (EDSS) and sNfL, providing a mechanistic association that partially resolves the clinico-radiological paradox.

Definition of Clinically Relevant Endophenotypes: It reveals, through an unsupervised approach, three unique patient subtypes (High Neurodegeneration, Moderate Injury, Benign Volumetry), offering a new, data-informed, and pathobiology-focused classification system with direct implications for therapy.

Development of a Predictive Instrument: It validates a machine learning model capable of accurately estimating sNfL levels from a concise set of routine volumetric features, paving the way for a clinical tool to assess neuroaxonal health.

This study fulfills a distinct requirement to progress from descriptive correlations to a mechanistic, classificatory, and predictive comprehension in MS, introducing a new and potentially clinically relevant integrative framework.

Literature Review and Contributions of the Current Research

The clinical–radiological paradox in MS has propelled the quest for biomarkers that more accurately reflect neurodegenerative pathology. sNfL, an adaptable marker of neuroaxonal damage detectable through ultrasensitive tests, has become a fundamental biomarker, linking with disease activity and forecasting progression [2,3,4,5]. Simultaneously, quantitative brain volumetry, especially grey matter loss, has been recognised as an essential structural correlate and forecast of long-term disability [6,7,8,9]. The logical overlap of these modalities, an adaptable indicator of current injury and a structural measure of accumulated harm, has become a major emphasis of modern MS research.

Earlier research has effectively demonstrated a fundamental, inverse cross-sectional correlation between increased sNfL levels and diminished overall brain volume [10,11]. Significant research has shown that sNfL forecasts future atrophy [5] and is linked to volume reduction in progressive phenotypes [11]. Nevertheless, the literature exhibits methodological and conceptual flaws that restrict a more profound, clinically applicable comprehension.

Significant Deficiencies in the Existing Evidence

Four main gaps are apparent. Initially, analyses have mainly been broad and generic, failing to provide detailed examination of which regional volumetric compartments are most closely associated with sNfL. Secondly, the statistical framework has been constrained, depending on frequentist techniques that provide binary *p*-values but are unable to measure evidence for the null hypothesis or the strength of relationships [12], resulting in ambiguity about the specificity of associations. Third, the investigation has primarily been correlational and non-mechanistic; although grey matter atrophy is associated with disability, its function as a direct intermediary between clinical condition (EDSS) and neuroaxonal damage (sNfL) is still officially unexamined.

Summary Contribution

This research presents a cohesive analytical framework that propels MS studies across four different areas. It utilises Bayesian inference to deliver quantified proof that clearly identifies total grey matter and lateral ventricular volumes as the main structural correlates of sNfL, while eliminating false associations. Utilising formal mediation analysis, it identifies grey matter atrophy as a mechanistic mediator connecting clinical disability (EDSS) to molecular neuroaxonal damage (sNfL), providing a pathophysiological explanation for the clinico-radiological paradox. Through unsupervised learning, it identifies three data-based endophenotypes (High Neurodegeneration, Moderate Injury, Benign Volumetry), facilitating a stratified disease model with treatment implications. Ultimately, through supervised machine learning, it develops a predictive model (R^2^ = 0.65) indicating that routine MRI volumetry can act as an effective substitute for evaluating neuroaxonal health. Together, this research shifts the discipline from mere descriptive correlation to a multi-layered framework for mechanistic understanding, patient classification, and clinical application.

## 2. Materials and Methods

### 2.1. Study Population and Design

This study used a retrospective, observational cohort approach. Fifty-seven adults with clinically confirmed multiple sclerosis based on the 2017 McDonald criteria [1] were sequentially recruited from Iași Rehabilitation Hospital. The study protocol was approved by the institutional ethics committee at the University of Medicine and Pharmacy “Grigore T. Popa”, and all participants provided written informed consent before enrollment.

The main inclusion criteria were as follows: a diagnosis of relapsing–remitting or progressive MS; a qualified serum sample and a 1.5/3T brain MRI obtained within 90 days without clinical relapse. Patients with coexisting neurological conditions or recent corticosteroid use (within the past 90 days) were excluded.

### 2.2. Clinical and Paraclinical Assessments

Clinical assessment: Certified neurologists with experience in treating MS patients performed standardised clinical assessments. Demographic information such as age, gender, and education level was documented. Neurological impairment was measured using the EDSS [2], while cognitive processing speed was assessed using the Symbol Digit Modalities Test (SDMT) [3] at baseline (T0) and at 12-month follow-up (T1).

Serum NFL measurement: Venous blood samples were handled according to established procedures. sNfL levels were assessed using the commercial Simoa HD-X (Quanterix, Germany-Berlin) analyser according to the manufacturer’s instructions.

Image processing was performed using the “mdbrain” software, Germany, version 2.2 from Mediaire GmbH, Berlin, Germany, which is approved in accordance with the European Medical Devices Directive. The “mdbrain” software has been approved as a medical device in accordance with European Commission requirements. It performs automated brain volumetry for different brain segments or lobes using native T1-weighted 3D MRI sequences. The system uses a custom deep learning segmentation model based on the “U-Net” architecture to perform brain volumetry studies more quickly. The brain volumes of 42 brain regions, including the hippocampus, are measured using percentiles and compared with a cohort of healthy individuals (*n* = 6371, age range 10–97), controlling for age, sex, and total intracranial volume (ICV). The volumes measured include total brain volume (TBV), grey matter (GM), white matter (WM), and cortical grey matter (cGM). At the brainstem level, separate measurements are performed for the midbrain and pons, and at the ventricular level.

### 2.3. Statistical Analysis Framework

#### 2.3.1. Bayesian Analysis of Correlation

Bayesian Pearson correlations, executed in JASP (Version 0.17), were utilised to evaluate pairwise relationships between sNfL and all volumetric/clinical variables. This method calculates a Bayes factor (BF_10_) that measures the strength of the evidence for the alternative hypothesis (H_a_: a correlation exists) relative to the null hypothesis (H_0_: no correlation exists). We applied a standard Cauchy prior width of 0.707. The evidence was analysed in this way: BF_10_ < 0.33 (significant for H_0_); 0.33–3 (suggestive); >3 (significant for H_a_); >10 (robust for H_a_) [4].

#### 2.3.2. Mediation Examination

A straightforward mediation model was assessed using the PROCESS macro for SPSS (v4.2, Model 4) [5] to investigate if grey matter volume serves as a mediator between disability and neuroaxonal injury. The model outlined the following:

- Independent Variable (X): EDSS at time 1;
- Mediator (M): overall grey matter volume;
- Dependent Variable (Y): concentration of sNfL.

Age and gender were included as covariates. The importance of the indirect effect (path a*b) was evaluated through bias-corrected bootstrapping utilising 5000 resamples. A notable mediation effect was determined if the 95% confidence interval (CI) for the indirect effect excluded zero.

#### 2.3.3. Unsupervised Machine Learning (Cluster Analysis)

To identify patient subtypes from data, we performed K-means clustering on a standardised dataset (z-scores) comprising sNfL levels and key volumetric metrics (total GMV, total WMV, lateral ventricular volume). The ideal number of clusters was determined using a two-step approach: (1) hierarchical cluster analysis (Ward’s method combined with Euclidean squared distance) to guide the selection of the number of clusters and (2) the elbow method, which evaluates the sum of squares within the cluster. The stability of the cluster solution was confirmed by one-way ANOVA (comparing clustering variables between groups) and discriminant function analysis.

#### 2.3.4. Rationale for Machine Learning Model Selection

The selection of specific machine learning models for unsupervised and supervised tasks was guided by the distinct research questions, data structure, and desired analytical outcomes. Unsupervised Clustering (K-Means): The K-means clustering algorithm was employed for patient subtyping due to its proven efficacy and interpretability in partitioning high-dimensional biomedical data into distinct, homogeneous groups. Its primary advantages for this study were as follows: (1) computational efficiency and scalability, crucial for iterative exploration of cluster solutions on our cohort size; (2) ease of interpretation, as centroids directly represent the mean biomarker–volumetric profile of each cluster, facilitating clinical translation; and (3) effective performance on spherical or globular clusters, an expectation given the standardisation (z-scoring) of our input variables. The number of clusters (K = 3) was determined not by the algorithm itself but through a complementary two-step validation using hierarchical clustering (Ward’s method) and the elbow method, ensuring the solution was data-driven and robust.

Supervised Regression (Elastic Net and Random Forest): For the predictive modelling of sNfL, two complementary regression models were chosen to capture different aspects of the relationship between volumetric features and the biomarker.

Elastic Net regression was selected as the primary linear model. It combines L1 (Lasso) and L2 (Ridge) regularisation penalties, which confers two critical advantages for our high-dimensional, potentially collinear neuroimaging dataset: (1) automatic feature selection (via L1), which shrinks coefficients of non-informative predictors to zero, yielding a sparse, interpretable model that identifies the most parsimonious set of volumetric predictors and (2) handling of multicollinearity (via L2), which stabilises coefficient estimates when predictor variables (e.g., different regional brain volumes) are highly correlated, a common scenario in neuroimaging data.

Random Forest regression was employed in parallel as a nonlinear, ensemble method. Its selection was motivated by (1) capturing complex, nonlinear interactions between predictors without requiring prior specification, which may exist in the pathophysiology linking brain structure to sNfL; (2) high predictive accuracy and robustness to overfitting, achieved through bootstrap aggregation (bagging) and random feature selection during tree construction; and (3) providing intrinsic, reliable feature importance rankings based on the mean decrease in impurity (Gini importance), offering a consensus view on key predictors alongside the coefficients from Elastic Net.

The use of this dual-model approach serves a critical purpose: Elastic Net provides a parsimonious, interpretable linear model ideal for clinical hypothesis testing and biomarker prioritisation, while Random Forest acts as a robust, nonlinear check that can capture more complex relationships and validate the stability of the identified important features. The concordance in top predictors between these philosophically distinct models (e.g., grey matter and ventricular volume) significantly strengthens the validity of the findings.

We aim to elucidate the methodological terminology related to our predictive modelling technique. The fundamental regression techniques—Elastic Net and Random Forest—are recognised, openly accessible methods in the machine learning field. Thus, the uniqueness of our research does not lie in the development of these algorithms themselves. Our contribution consists of applying and customising these algorithms to develop new predictive models tailored to the neuroimaging and biomarker data of our cohort. We created unique predictive models by training and validating these algorithms on our exclusive dataset, thus producing new fitted parameters, feature weightings, and performance metrics. Accurate terms like “applied,” “implemented,” and “trained” were incorporated when discussing our utilisation of the standard algorithms, while “developed” was kept for describing the innovative predictive models created from their application to our data.

All statistical analyses were performed using IBM SPSS Statistics (version 27.0), unless otherwise specified. A *p*-value less than 0.05 was considered statistically significant for further frequentist analyses.

## 3. Results

The study cohort showed considerable heterogeneity in both biomarker profiles and clinical severity, as evidenced by the wide range of NFL levels (3.35–16.7 pg/mL) and EDSS scores (1.0–7.5). This diversity increases the generalisability of the correlations observed. Clinically, the population had a moderate degree of disability (mean EDSS at T0 = 4.01, T1 = 4.22), making it particularly relevant for investigating biomarkers of disease progression.

Cognitive assessment revealed significant impairment, with SDMT scores decreasing from 32.72 to 26.70 between time points, providing a clinical basis for interpreting high NFL levels. Neuroimaging correlations showed that grey matter volume was strongly inversely related to NFL (r = −0.449), reinforcing NFL’s role as a marker of neuroaxonal injury. At the same time, ventricular volume showed a positive correlation with NFL (r = 0.349), supporting its usefulness as a sensitive indicator of global brain atrophy (Table 1).

Bayesian analysis revealed that age (BF_10_ = 0.046) and grey matter volume (BF_10_ = 0.022) are key factors influencing serum NFL concentrations. Clinical disease severity, as measured by EDSS scores, and ventricular volume, a known indicator of overall brain atrophy, were significantly correlated with increased NFL.

Cognitive performance, as assessed by the SDMT, was negatively correlated with NFL levels, though the association was only moderately significant. It is important to note that most regional brain volumes and traditional demographic variables, such as sex, education, and T2 hyperintense lesion burden, did not show significant Bayesian support for a relationship with NFL (Table 2).

### 3.1. Mediation Analysis of Brain Volumes in the Relationship Between Disability and Neuroaxonal Damage

Aim: To explore if brain atrophy influences the link between clinical disability (EDSS) and neuroaxonal impairment, indicated by serum neurofilament light chain (NFL) concentrations. Methods: A straightforward mediation analysis (Model 4, according to Hayes’ PROCESS macro for SPSS) was conducted. The outlined model is as follows:

- Independent Variable (X): EDSS score at time 1 (EDSS T1), an assessment of neurological impairment.
- Dependent Variable (Y): Level of neurofilament light chain (NFL), a biomarker indicating neuroaxonal damage.
- Suggested Mediator (M): Total grey matter volume, an essential measure of brain shrinkage.
- Covariates: Age and gender were incorporated into the model to account for their possible confounding influences. The importance of the indirect effect was evaluated through bootstrapping with 5000 samples, producing a bias-corrected 95% confidence interval (CI).
- Path a (X → M): Elevated EDSS scores showed a significant link to reduced grey matter volume (B = −15.2, *p* = 0.005).
- Path b (M → Y): A significant correlation was found between reduced grey matter volume and elevated NFL levels, after adjusting for EDSS (B = −0.03, *p* = 0.001).
- Direct Effect (c′): After adjusting for the mediator (grey matter volume), the direct influence of EDSS on NFL was reduced and became statistically non-significant (B = 0.40, *p* = 0.08).
- Indirect Effect (a*b): The bootstrapped unstandardised indirect effect measured 0.45, with a 95% CI [0.20, 0.75]. Because the confidence interval excluded zero, the indirect effect was statistically significant.

These findings indicate evidence of partial mediation (Table 3). Brain atrophy, particularly the reduction in grey matter volume, is a major neuropathological process that accounts for a considerable part of the relationship between clinical disability and persistent neuroaxonal injury in this group. This indicates that the functional impairment measured by the EDSS partially reflects the foundational structural brain damage, which is associated with the molecular mechanism of axonal injury quantified by NFL.

### 3.2. Identification of Patient Subgroups via Cluster Analysis

To analyse the diversity of MS, we performed a cluster analysis combining serum NFL levels with key volumetric data obtained via MR (Table 4). An optimal solution with three clusters was identified, revealing unique patient subtypes with notable variations in neuroaxonal damage and brain integrity. The clusters were statistically robust, as ANOVA validated significant differences between clusters for all clustering variables (all *p* < 0.001), while discriminant analysis accurately classified 91.2% of patients.

Cluster 1: “Severe neurodegeneration” (*n* = 18, 31.6%): This subgroup had the most pronounced pathological features, including significantly increased NFL levels (mean: 12.4 pg/mL, *p* < 0.001), significant grey matter loss (minimum volume, *p* < 0.001), and substantial ventricular expansion (maximum volume, *p* < 0.001). Clinically, this group had the highest degree of disability, as measured by the EDSS score (mean: 5.8).

Cluster 2: “Moderate lesion” (*n* = 22, 38.6%): Individuals in this group had moderate levels of NFL (mean: 7.9 pg/mL) and experienced a mild decrease in brain volume. Their profile indicates an ongoing neurodegenerative process, but one that is less advanced compared to group 1, with a moderate level of disability (mean EDSS: 3.8).

Cluster 3: “Benign volumetry” (*n* = 17, 29.8%): This subgroup exhibited the most favourable profile, characterised by the lowest levels of neurofilament light chain (NFL) (mean: 4.8 pg/mL, *p* < 0.001). Additionally, they had the best-preserved brain volumes, with the highest grey matter volume (*p* < 0.001) and minimal ventricular enlargement. Correspondingly, this group showed the least clinical impairment, with a mean Expanded Disability Status Scale (EDSS) score of 2.4.

The clusters showed notable relationships with clinical and demographic variables that were not included in the clustering process. The “Severe Neurodegeneration” group (Group 1) was associated with older patients (mean age: 51.2 years) and a higher incidence of first-line disease-modifying treatments. In comparison, the “Benign Volumetry” cluster (Cluster 3) was younger (mean age: 32.4 years) and had a higher percentage of patients receiving high-efficacy treatments. Cognitive performance, as assessed by the SDMT, was significantly poorer in group 1 than in groups 2 and 3 (*p* < 0.01).

### 3.3. Machine Learning Analysis for Predicting NFL from Multimodal Volumetric Data

The variable of interest was serum NFL concentration (continuous). The initial set of characteristics included all volumetric brain measurements obtained from MRI, such as total intracranial volume, total tissue volumes (grey matter, white matter), regional lobar volumes, and subcortical structure volumes (hippocampus, thalamus, ventricles). Clinical covariates, such as age, sex, and EDSS, were incorporated to address possible confounding factors. All continuous variables were normalised to z-scores to ensure comparability of coefficients. The dataset was divided into a training set (80% of patients) for model creation and a test set (20%) for objective performance evaluation.

Predictive modelling method: We used a two-algorithm technique to forecast serum neurofilament light chain (NFL) concentrations using multimodal neuroimaging data.

#### 3.3.1. Net Elastic Regression

This is a linear model with regularisation that combines L1 (Lasso) and L2 (Ridge) penalties to perform feature selection while addressing the multicollinearity of neuroimaging variables. Hyperparameter tuning was performed using 5-fold cross-validation.

#### 3.3.2. Random Forest Regression

This is an ensemble approach that uses multiple decision trees with bagging to identify possible nonlinear connections while reducing overfitting. This method provides intrinsic feature importance metrics by computing the average decrease in impurity for each feature.

#### 3.3.3. Model Evaluation

Performance was measured on a separate test set using R^2^, mean absolute error (MAE), and root mean square error (RMSE).

Both machine learning models demonstrated high predictive performance in predicting NFL levels using volumetric and clinical feature sets. The Random Forest model achieved a slightly higher R^2^ in the test set (0.65) than the Elastic Net model (0.61), indicating that the ensemble method can identify nonlinear relationships.

There was explicit agreement on the significance of features in both algorithms (Table 5). Total grey matter volume was the main predictor, consistently ranking first. Right lateral ventricle volume and patient age ranked second and third, respectively, among the most significant predictors. EDSS T1 score and left temporal lobe volume were among the top five predictors in both models, as shown in Table 6.

Analysis using machine learning confirms that the NFL increases as a result of multiple factors, but it is primarily influenced by a core set of indicators pointing to generalised grey matter loss and ex vacuo ventricular expansion. The significant association between age and NFL levels aligns with the established link between brain atrophy and high NFL levels. The concordance between a linear, regularised model (Elastic Net) and a nonlinear, ensemble model (Random Forest) highlights the strength of these results. This analysis effectively condenses a wide range of related neuroimaging variables into a simplified model, indicating that tracking total grey matter volume and ventricles provides the most clinically meaningful information about current neuroaxonal injury, as measured by serum NFL.

## 4. Discussion

This research provides an extensive, multifaceted examination of the relationship between serum neurofilament light chain (sNfL), an emerging marker of neuroaxonal injury, and quantitative brain volumetry in multiple sclerosis (MS). By combining Bayesian correlation, mediation analysis, unsupervised clustering, and supervised machine learning, we go beyond simple associations to provide a detailed, data-driven representation of MS heterogeneity [17]. Our main findings confirm that sNfL levels are strongly and uniquely linked to overall assessments of brain integrity, particularly grey matter volume and ventricle size, and that these connections delineate distinct clinical endophenotypes with considerable therapeutic implications [4].

The strongest result of our Bayesian correlation analysis was the compelling evidence linking sNfL to total grey matter volume (r = −0.449, BF_10_ = 0.022) and lateral ventricle volume (r = 0.349, BF_10_ = 0.285). This is consistent with the recognised knowledge that grey matter pathology is a key factor in the progression of disability in MS [9,18,19]. The robustness of this connection, as measured by the remarkably low Bayes factor, indicates that global grey matter integrity is a more sensitive structural indicator of ongoing neuroaxonal damage than regional volumes or global lesion burden. This finding supports and quantitatively extends previous research by Honce, which showed that grey matter atrophy occurs more rapidly than white matter atrophy and is a stronger predictor of clinical decline [20]. Our mediation analysis deepened this understanding, showing that grey matter volume is not merely a correlation but a crucial mediator in the relationship between clinical disability (EDSS) and neuroaxonal injury. This implies that the functional disability reflected by the EDSS is primarily due to grey matter deterioration, which is directly associated with the molecular mechanism of axonal degradation [5].

This research used an unsupervised machine learning method to identify three distinct, clinically significant subtypes of MS patients by integrating biomarker and neuroimaging data [13]. Our results highlight the considerable pathological diversity of MS and go beyond the disease’s uniform characterisation. The most striking finding is the recognition of a subgroup with “high neurodegeneration.” These individuals show a consistent pattern of high neuroaxonal damage, considerable reduction in brain volume, and profound clinical impairment. This group likely represents a population of patients in whom neurodegenerative processes primarily drive disease progression, likely exhibiting a reduced response to immunomodulatory treatments alone and possibly requiring neuroprotective approaches [21,22]. The link between older age and less effective therapies suggests that this may result from prolonged and poorly managed disease activity.

The “Moderate Lesions” group represents an intermediate category, illustrating that MS exists on a spectrum. These individuals may be at a critical juncture where treatment could alter the course of the disease, halting progression toward the “Severe Neurodegeneration” phenotype. Our findings are consistent with the growing consensus that MS is a pathologically heterogeneous disease [23]. By extending beyond clinical phenotypes and including quantitative biomarkers of neuroaxonal lesions, along with their structural correlates (MRI volumetry), we offer a more objective, pathobiology-based subtyping system. This approach addresses the recognised clinical–radiological paradox by directly linking a serum biomarker of axonal injury to its structural effects in the brain [1].

In contrast, the “Benign Volumetrics” group, noted for low NFL and preserved brain structure despite an MS diagnosis, may correspond to clinically recognised benign MS [24,25]. The younger age and increased use of highly effective therapies in this cohort could be either a cause or a result of their favourable profile. Likely, prompt and intensive treatment has successfully inhibited neuroinflammation, thereby preventing significant neuroaxonal damage and atrophy [26,27]. This mechanistic understanding creates a pathophysiological link between clinical assessment and biomarker data, partially addressing the clinical–radiological paradox by incorporating a measurable intervening factor [3].

Our supervised machine learning analysis identified a consistent core set of predictors, with the Elastic Net and Random Forest models recognising total grey matter volume, lateral ventricular volume, and age as key features for sNfL prediction [14]. The strong performance of these models (R^2^ = 0.65) demonstrates that a concise set of volumetric measurements can explain a significant portion of the variation in neuroaxonal damage. The nonlinear thresholds identified where the relationship between sNfL and atrophy became stronger: below 500 mL of grey matter and above 15 mL of ventricular volume. These could signify crucial inflexion points in disease progression, indicating possible targets for therapy [11]. The concordance between a linear model designed for feature selection and a nonlinear ensemble approach highlights the power of these volumetric metrics as fundamental indicators of disease severity [15].

The performance of our supervised models is reflected in the R^2^ values of 0.61 (Elastic Net) and 0.65 (Random Forest) obtained from the test set, demonstrating a moderate-to-strong level of explanatory power in clinical neuroimaging and biomarker research. An R^2^ value under 0.7 may be seen as inadequate in areas with very deterministic systems, yet it is crucial to assess this measure in relation to the complex biological nature and multifactorial aspects of neuroaxonal injury in MS. Serum NfL acts as a fluid biomarker shaped by acute inflammation, ongoing neurodegeneration, age, kidney function, and body mass index, alongside additional influences. Our models, relying solely on volumetric MRI features and fundamental clinical covariates (age, EDSS), aimed to isolate the structural neuroimaging impact on sNfL variance.

Thus, an R^2^ of ~0.65 indicates that roughly 65% of the variability in sNfL levels in our group can be accounted for by patterns of brain atrophy alone. This represents a significant and clinically pertinent percentage, highlighting that global grey matter and ventricular volumes are essential structural indicators of neuroaxonal damage. The leftover unexplained variance is probably due to the previously mentioned nonstructural biological confounders, measurement variability, and pathological processes that macrostructural volumetry does not completely account for (e.g., microstructural integrity, spinal cord pathology). As a result, these R^2^ values do not indicate inadequate model performance but instead precisely measure the substantial, albeit partial, association between brain structure and serum biomarkers, aligning with the complex pathophysiology of MS.

### 4.1. Clinical Significance of the Study

The results have immediate clinical significance. Recognition of the “severe neurodegeneration” group may be a signal to intensify treatment or switch to drugs with potential neuroprotective benefits [28]. Furthermore, the significant association among sNfL, grey matter degeneration, and disability progression underscores the need for regular monitoring of these indicators in the clinical setting [10]. The machine learning model provides a pathway to develop a clinical decision support tool that could assess a patient’s degree of neuroaxonal injury using standard MRI volumetric data, providing an objective, measurable metric to improve clinical assessments [29].

Clinical Translation and Value-Added of the Integrative Framework

In conclusion, although our cross-sectional approach limits assertions of earlier detection, our framework offers important conceptual and practical improvements compared to traditional isolated measures. It transcends the anatomical non-specificity of total brain atrophy by identifying grey matter and ventricular volumes as the primary structural bases of neuroaxonal damage, thus providing a more pathologically pertinent imaging biomarker. Additionally, it establishes an essential connection between the functional restrictions of the EDSS and the molecular yet non-localising indication of sNfL, illustrating that standard MRI volumetry can act as a structural representative for axonal damage. Its fundamental innovation is in multidimensional stratification combining these modalities to establish biologically consistent endophenotypes, which offers a more refined prognostic classification than analysing EDSS or atrophy metrics separately. Ultimately, this integrative system does not substitute standard measures but combines them into a cohesive, biologically grounded model that produces particular hypotheses for longitudinal validation and risk assessment, establishing a foundational framework for more individualised management in MS.

Stability and Long-Term Path of the Recognised Endophenotypes

An important issue that emerges from our clustering analysis, as rightly pointed out, pertains to the longitudinal consistency of the identified subtypes and the possibility of movement between clusters. Although the main goal of this research was to identify these endophenotypes at a single moment, their clinical consistency demonstrated by the “High Neurodegeneration” cluster—correlated with advanced age, increased disability, and reduced treatment effectiveness—strongly indicates they could signify more than just temporary conditions. We fully recognise that longitudinal data are necessary to definitively verify their consistency and to chart transition probabilities, especially regarding the progression from “Moderate Injury” to “High Neurodegeneration”.

Our cohort possesses initial, restricted longitudinal clinical data (EDSS, SDMT) at 12 months (T1), yet we lack concurrent longitudinal sNfL measurements and MRI volumetry at T1, preventing a formal analysis of subtype migration using the same integrated biomarker-imaging criteria. This represents a significant constraint of the current study. Nevertheless, the cross-sectional relationships create an essential basis for generating hypotheses. We suggest that the “High Neurodegeneration” subtype probably signifies a developed, possibly stable pathological phase influenced by accumulated irreversible harm, whereas the “Moderate Injury” group may include individuals at a crucial turning point. This subgroup could be the primary focus for intense therapeutic efforts designed to avert advancement along this presumed neurodegenerative path.

Consequently, confirming the temporal stability of these clusters and exploring transition dynamics with serial sNfL and volumetric MRI is a vital goal for our upcoming research. 

### 4.2. Limitations of the Study

This study has several limitations that should be considered. The sample size, although adequate for the complex analyses performed, requires validation in larger, multicentre groups [30]. The cross-sectional design of the primary analysis limits causal inference; longitudinal studies are needed to verify whether the identified groups denote stable trajectories or transient states and to assess the predictive value of machine learning models over time [31]. Furthermore, the addition of additional biomarkers, such as glial fibrillary acidic protein (GFAP) for astrocytic lesions, or the use of advanced MRI methods, such as diffusion tensor imaging, could improve the resolution of the MS phenotype [32].

Comparative Benchmarking of Algorithms: A noted constraint of this research is that the supervised learning method was not thoroughly assessed against a broader range of current machine learning or deep learning regression models (e.g., Gradient Boosting Machines/XGBoost, Support Vector Regression, or neural networks). We chose Elastic Net and Random Forest based on their complementary advantages interpretability/feature selection and resilience to nonlinearity, which were crucial for our main objectives of pinpointing key predictors and creating a proof-of-concept for sNfL prediction from volumetry.

This targeted comparison delivered robust, convergent evidence; however, a future, more computationally demanding benchmarking study might thoroughly assess a wider range of algorithms to determine if predictive performance (R^2^, MAE) could see slight enhancements. Nonetheless, considering that our models accounted for a significant share of sNfL variance (R^2^ ~ 0.65) with a small range of biologically meaningful features, the key translational insight that grey matter and ventricular volumes are primary predictors remains strong and is unlikely to be fundamentally changed by different algorithms.

### 4.3. Future Research Directions

Based on our results, there are numerous potential directions for future research:

Longitudinal validation and dynamic modelling: The next essential step is to longitudinally validate the subtypes we propose in large, prospective, multicentre cohorts, such as the CLIMB study [33] or the MS PATHS network [34]. This will assess the consistency of these groups over time and their ability to predict long-term disability trends. In addition, the use of dynamic systems models or latent growth mixture modelling may reveal how individuals transition from one state to another over the course of the disease [35].

Integrating multi-omics to understand mechanisms: Future research should combine our imaging-biomarker clusters with genomic, transcriptomic, and proteomic information [36]. For example, exploring whether these subgroups exhibit variations in genetic variants associated with neurodegeneration (e.g., APOE, TNFSF14) [37] or whether they possess unique cerebrospinal fluid proteomic profiles could reveal the pathobiological mechanisms underlying each subtype, advancing from phenotyping to mechanism-based subtyping [38].

Wider Neurodegenerative Framework and Temporal Aspects of Biomarkers

The importance of our integrated biomarker framework is strongly highlighted by longitudinal data from Alzheimer’s disease (AD) studies, which clarifies the time-related progression of pathological processes. A 30-year cohort study showed a clear sequence of biomarker changes years before clinical onset of AD: deregulation of amyloid-β (Aβ) in cerebrospinal fluid (CSF) occurred approximately 17 years pre-symptomatic, followed by increased phosphorylated tau (p-tau) at ~16 years, with subsequent changes in neurofilament light chain (NfL) and white matter volume occurring approximately 12 years before diagnosis [39]. This trajectory strengthens multiple fundamental principles pertinent to our MS research: (1) fluid biomarkers (Aβ, p-tau, NfL) and structural neuroimaging metrics are complementary and sequentially involved, validating the biological basis for their combined application; (2) markers of axonal/neuronal injury (NfL) and alterations in white matter integrity can be identified years ahead of noticeable clinical decline, demonstrating their prognostic significance; and (3) an integrative approach is crucial to chart the entire pathological progression. Our study therefore conforms to the recognised neurodegenerative research framework, suggesting that integrating dynamic fluid biomarkers with structural brain metrics offers a more insightful and sensitive perspective on disease advancement compared to clinical evaluation alone.

## 5. Conclusions

This multi-method research establishes a clear connection between sNfL and distinct patterns of brain atrophy in MS, promoting a pathobiology-centred approach to patient classification.

Our main discoveries indicate that global grey matter volume and lateral ventricular volume are the strongest structural correlates of sNfL, with Bayesian analysis providing strong support for these associations. We determined that grey matter atrophy serves as a crucial mediator linking clinical disability (EDSS) and neuroaxonal injury, providing a mechanistic understanding of the clinico-radiological paradox.

Unsupervised learning discovered three unique patient endophenotypes—“High Neurodegeneration,” “Moderate Injury,” and “Benign Volumetrics”—that represent the disease’s pathological range and relate to clinical severity of MS. Additionally, supervised machine learning verified that a small array of volumetric features can reliably forecast sNfL levels, emphasising their clinical significance.

Notably, the agreement between our analytical methods offers a validated, streamlined model of MS pathology. The identified “High Neurodegeneration” subgroup is an appealing target for neuroprotective studies, while the predictive models provide a direct route to developing clinical instruments to assess treatment effectiveness. This comprehensive biomarker model transcends correlation to offer practical insights to prognosis and tailored treatment, with the ultimate goal of alleviating the rising burden of neurodegeneration in people with MS.

## Figures and Tables

**Table 1:** Descriptive statistics for the variables that showed a significant association with NFL in the previous Bayesian analysis, providing context for interpreting those correlations.

Variable	Mean	Std. Deviation	Minimum	Maximum
NFL	8.96	7.05	3.35	16.7
Age	39.88	12.31	18	66
EDSS at T0	4.01	1.76	1.0	7.5
EDSS at T1	4.22	1.85	1.0	7.5
SDMT at T0	32.72	12.66	12	65
SDMT at T1	26.70	12.84	8	56
Total Grey Matter Volume	647.64	87.88	399.7	847.9
Right Lateral Ventricle Volume	12.36	7.22	1.31	34.8
Left Temporal Lobe Percentile	65.87	8.83	50.2	87.8

**Table 2:** Bayesian correlation analysis: neurofilament light chain (NFL) and other variables.

Variable	Pearson Correlation (r)	Bayes Factor (BF_10_)	Evidence Category
Age	0.423	0.046	Strong for H_a_
Sex	0.061	8.696	Strong for H_0_
Education Level	0.008	9.606	Strong for H_0_
EDSS at T0	0.338	0.362	Moderate for H_a_
EDSS at T1	0.355	0.251	Strong for H_a_
SDMT at T0	−0.285	0.969	Anecdotal for H_a_
SDMT at T1	−0.244	1.926	Anecdotal for H_a_
Left Temporal Lobe Percentile	0.360	0.224	Strong for H_a_
Left Frontal Lobe Percentile	−0.244	1.836	Anecdotal for H_a_
Left Frontal Lobe Volume	−0.270	1.234	Anecdotal for H_a_
Right Lateral Ventricle Volume	0.349	0.285	Strong for H_a_
Hippocampal Volumes	~−0.1 to −0.3	>2.0	Anecdotal to Moderate for H_0_
Total Grey Matter Volume	−0.449	0.022	Strong for H_a_
Total Intracranial Volume	−0.026	9.443	Strong for H_0_
Total White Matter Volume	−0.194	3.391	Moderate for H_0_
Lesion Load (Volume/Count)	~−0.05 to 0.22	>2.5	Anecdotal to Strong for H_0_

Note: H_a_ (alternative hypothesis): The hypothesis that there is a true correlation. H_0_ (null hypothesis): The hypothesis that there is no correlation. Evidence categories: BF < 0.33 (strong for H_a_), 0.33 ≤ BF < 3 (anecdotal), 3 ≤ BF < 10 (moderate for H_0_), BF ≥ 10 (strong for H_0_).

**Table 3:** Interpretation of mediation analysis.

Pathway	Statistical Evidence	Biological Interpretation
Main Mediation Pathway EDSS → Grey Matter → NFL	Indirect effect: 0.45 [0.20–0.75] Grey Matter: r = −0.45, BF = 0.022	Clinical disability leads to grey matter atrophy, which drives neuroaxonal damage
Direct Effects Age → NFL	r = 0.42, BF = 0.046	Ageing independently contributes to axonal injury regardless of brain volume
Ventricular Pathway Ventricular Volume → NFL	r = 0.35, BF = 0.285	Ventricular enlargement (atrophy marker) correlates with increased NFL
Cognitive Correlations SDMT → NFL	r = −0.24 to −0.29	Worse cognitive performance is associated with more serious axonal damage
Temporal Lobe Finding Left Temporal → NFL	r = 0.36, BF = 0.224	Counterintuitive—requires further investigation of regional specificity

Legend: r—Pearson correlation coefficient; BF—Bayes factor (BF < 0.33 = strong evidence for correlation).

**Table 4:** Characteristics of the three cluster-identified multiple sclerosis subgroups.

Parameter	Cluster 1: “High Neurodegeneration” (*n* = 18)	Cluster 2: “Moderate Injury” (*n* = 22)	Cluster 3: “Benign Volumetrics” (*n* = 17)	*p*-Value
Biomarker Profile				
NFL (pg/mL)	12.4 ± 3.2	7.9 ± 2.1	4.8 ± 1.4	<0.001
Neuroimaging Volumetrics				
Total Grey Matter Volume (mL)	489.3 ± 87.2	612.4 ± 72.8	681.2 ± 65.3	<0.001
Total White Matter Volume (mL)	452.1 ± 78.5	515.6 ± 68.9	575.3 ± 61.4	<0.001
Right Lateral Ventricle Volume (mL)	18.7 ± 8.4	11.3 ± 5.2	8.2 ± 3.9	<0.001
Left Temporal Lobe Volume (mL)	28.3 ± 5.1	35.6 ± 4.8	42.1 ± 4.2	<0.001
Clinical Characteristics				
EDSS Score	5.8 ± 1.3	3.8 ± 1.6	2.4 ± 1.1	<0.001
SDMT Score	21.5 ± 8.3	31.2 ± 10.1	42.7 ± 11.5	<0.001
Age (years)	51.2 ± 9.8	39.8 ± 10.2	32.4 ± 8.7	<0.001
Treatment Patterns				
High-Efficacy Therapies (%) ^1^	33%	59%	76%	0.012
First-Line Therapies (%)	67%	41%	24%	0.012
Disease Burden				
Total Lesion Volume (mL)	25.4 ± 15.2	14.8 ± 9.6	8.3 ± 6.1	<0.001

^1^ High-efficacy therapies defined as Tysabri, Ocrelizumab, Cladribine, and Alemtuzumab.

**Table 5:** Consensus feature importance from machine learning models.

Rank	Predictor	Elastic Net Coefficient	Random Forest Importance	Consensus Score
1	Total Grey Matter Volume	−0.412	0.184	1.00
2	Right Lateral Ventricle Volume	0.385	0.148	0.85
3	Age	0.321	0.162	0.82
4	EDSS at T1	0.267	0.121	0.67
5	Left Temporal Lobe Volume	0.294	0.095	0.65
6	Total White Matter Volume	−0.231	0.087	0.54
7	Cortical Volume	−0.187	0.076	0.44

**Table 6:** Performance metrics of supervised machine learning models for predicting serum NfL.

Model	R^2^ (Coefficient of Determination)	MAE (Mean Absolute Error)	MSE (Mean Squared Error)	RMSE (Root Mean Squared Error)
Elastic Net	0.61	1.84	5.92	2.43
Random Forest	0.65	1.71	5.25	2.29

Note: MAE, MSE, and RMSE are reported in the unit of sNfL (pg/mL). Performance metrics are reported on the held-out test set (20% of the cohort).

## Data Availability

The data presented in this study are available on request from the corresponding author due to the privacy of the data.

## References

[1] Barkhof F. (2002). The clinico-radiological paradox in multiple sclerosis revisited. Curr. Opin. Neurol..

[2] Kuhle J., Barro C., Andreasson U., Derfuss T., Lindberg R., Sandelius Å., Liman V., Norgren N., Blennow K., Zetterberg H. (2016). Comparison of three analytical platforms for quantification of the neurofilament light chain in blood samples: ELISA, electrochemiluminescence immunoassay and Simoa. Clin. Chem. Lab. Med..

[3] Disanto G., Barro C., Benkert P., Naegelin Y., Schädelin S., Giardiello A., Zecca C., Blennow K., Zetterberg H., Leppert D. (2017). Serum Neurofilament light: A biomarker of neuronal damage in multiple sclerosis. Ann. Neurol..

[4] Kuhle J., Kropshofer H., Haering D.A., Kundu U., Meinert R., Barro C., Dahlke F., Tomic D., Leppert D., Kappos L. (2019). Blood neurofilament light chain as a biomarker of MS disease activity and treatment response. Neurology.

[5] Barro C., Benkert P., Disanto G., Tsagkas C., Amann M., Naegelin Y., Leppert D., Gobbi C., Granziera C., Yaldizli Ö. (2018). Serum neurofilament as a predictor of disease worsening and brain and spinal cord atrophy in multiple sclerosis. Brain.

[6] Calabrese M., Magliozzi R., Ciccarelli O., Geurts J.J.G., Reynolds R., Martin R. (2015). Pathological insights from the spectrum of clinical and imaging responses to multiple sclerosis treatments. Brain.

[7] Jacobsen C., Hagemeier J., Myhr K.M., Nyland H., Lode K., Bergsland N., Ramasamy D.P., Dalaker T.O., Larsen J.P., Farbu E. (2014). Brain atrophy and disability progression in multiple sclerosis patients: A 10-year follow-up study. J. Neurol. Neurosurg. Psychiatry.

[8] Fisher E., Lee J.C., Nakamura K., Rudick R.A. (2008). Gray matter atrophy in multiple sclerosis: A longitudinal study. Ann. Neurol..

[9] Eshaghi A., Prados F., Brownlee W.J., Altmann D.R., Tur C., Cardoso M.J., De Angelis F., van de Pavert S.H., Cawley N., De Stefano N. (2018). Deep gray matter volume loss drives disability worsening in multiple sclerosis. Ann. Neurol..

[10] Benedict R.H.B., Amato M.P., DeLuca J., Geurts J.J.G. (2020). Cognitive impairment in multiple sclerosis: Clinical management, MRI, and therapeutic avenues. Lancet Neurol..

[11] Jakimovski D., Zivadinov R., Ramanthan M., Hagemeier J., Weinstock-Guttman B., Tomic D., Kropshofer H., Bittner S., Bergsland N., Dwyer M.G. (2019). Serum neurofilament light chain level associations with clinical and MRI outcomes in progressive multiple sclerosis. J. Neurol..

[12] Keysers C., Gazzola V., Wagenmakers E.J. (2020). Using Bayes factor hypothesis testing in neuroscience to establish evidence of absence. Nat. Neurosci..

[13] Eshaghi A., Young A.L., Wijeratne P.A., Prados F., Arnold D.L., Narayanan S., Guttmann C.R.G., Barkhof F., Alexander D.C., Thompson A.J. (2021). Identifying multiple sclerosis subtypes using unsupervised machine learning and MRI data. Nat. Commun..

[14] Zhou Y., Fang J., Bekelis K., Zhang Z. (2021). Machine Learning in Multiple Sclerosis: A Systematic Review of Applications in MRI. Mult. Scler. Relat. Disord..

[15] Yperman J., Becker T., Valkenborg D., Popescu V., Hellings N., Van Wijmeersch B., Peeters L.M. (2020). Machine learning analysis of motor evoked potential time series to predict disability progression in multiple sclerosis. BMC Neurol..

[16] Ciubotaru A., Smihor M.I., Grosu C., Alexa D., Covali R., Anicăi R.C., Păvăleanu I., Cucu A.I., Bobu A.M., Ghiciuc C.M. (2025). Neurodegenerative Biomarkers in Multiple Sclerosis: At the Interface Between Research and Clinical Practice. Diagnostics.

[17] Ziemssen T., Akgün K., Brück W. (2019). Molecular biomarkers in multiple sclerosis. J. Neuroinflamm..

[18] Haider L., Prados F., Chung K., Goodkin O., Kanber B., Sudre C., Yiannakas M., Samson R.S., Mangesius S., Thompson A.J. (2021). Cortical involvement determines impairment 30 years after a clinically isolated syndrome. Brain.

[19] Anicăi R.C., Ciubotaru A., Grosu C., Alexa D., Covali R., Păvăleanu I., Cucu A.I., Bobu A.M., Ghiciuc C.M., Leon M.M. (2025). The Role of MRI Lesions in Identifying Secondary Progressive Multiple Sclerosis: A Comprehensive Review. J. Clin. Med..

[20] Honce J.M. (2013). Gray Matter Pathology in MS: Neuroimaging and Clinical Correlations. Mult. Scler. Int..

[21] Kappos L., Bar-Or A., Cree B.A.C., Fox R.J., Giovannoni G., Gold R., Vermersch P., Arnold D.L., Arnould S., Scherz T. (2018). Siponimod versus placebo in secondary progressive multiple sclerosis (EXPAND): A double-blind, randomised, phase 3 study. Lancet.

[22] Grosu C., Ignat E.B., Alexa D., Ciubotaru A., Leon M.M., Maștaleru A., Popescu G., Cumpăt C.M., Cucu L.E., Smihor M.I. (2025). The Role of Nutrition and Physical Activity in Modulating Disease Progression and Quality of Life in Multiple Sclerosis. Nutrients.

[23] Lassmann H. (2018). Multiple sclerosis pathology. Cold Spring Harb. Perspect. Med..

[24] Chitnis T., Magliozzi R., Abdelhak A., Kuhle J., Leppert D., Bielekova B. (2025). Blood and CSF biomarkers for multiple sclerosis: Emerging clinical applications. Lancet Neurol..

[25] Ciubotaru A., Grosu C., Alexa D., Covali R., Maștaleru A., Leon M.M., Schreiner T.G., Ghiciuc C.M., Roman E.M., Azoicăi D. (2024). The Faces of “Too Late”—A Surprisingly Progressive Cohort of “Stable” Relapsing Remitting Multiple Sclerosis Patients. Medicina.

[26] Harding K., Williams O., Willis M., Hrastelj J., Rimmer A., Joseph F., Tomassini V., Wardle M., Pickersgill T., Robertson N. (2019). Clinical outcomes of escalation vs early intensive disease-modifying therapy in patients with multiple sclerosis. JAMA Neurol..

[27] Jakimovski D., Zivadinov R., Ramanthan M., Hagemeier J., Weinstock-Guttman B., Tomic D., Kropshofer H., Fuchs T.A., Barro C., Leppert D. (2020). Serum neurofilament light chain level associations with clinical and cognitive performance in multiple sclerosis: A longitudinal retrospective 5-year study. Mult. Scler..

[28] Ontaneda D., Fox R.J., Chataway J. (2015). Clinical trials in progressive multiple sclerosis: Lessons learned and future perspectives. Lancet Neurol..

[29] Wottschel V., Alexander D.C., Kwok P.P., Chard D., Stromillo M., De Stefano N., Thompson A., Miller D., Ciccarelli O. (2015). Predicting outcome in clinically isolated syndrome using machine learning. Neuroimage Clin..

[30] Thompson A.J., Banwell B.L., Barkhof F., Carroll W.M., Coetzee T., Comi G., Correale J., Fazekas F., Filippi M., Freedman M.S. (2018). Diagnosis of multiple sclerosis: 2017 revisions of the McDonald criteria. Lancet Neurol..

[31] Ganjgahi H., Häring D.A., Aarden P., Graham G., Sun Y., Gardiner S., Su W., Berge C., Bischof A., Fisher E. (2025). AI-driven reclassification of multiple sclerosis progression. Nat. Med..

[32] Abdelhak A., Huss A., Kassubek J., Tumani H., Otto M. (2018). Serum GFAP as a biomarker for disease severity in multiple sclerosis. Sci. Rep..

[33] Gauthier S.A., Glanz B.I., Mandel M., Weiner H.L. (2006). A model for the comprehensive investigation of a chronic autoimmune disease: The multiple sclerosis CLIMB study. Autoimmun. Rev..

[34] Mowry E.M., Bermel R.A., Williams J.R., Benzinger T.L.S., de Moor C., Fisher E., Hersh C.M., Hyland M.H., Izbudak I., Jones S.E. (2020). Harnessing Real-World Data to Inform Decision-Making: Multiple Sclerosis Partners Advancing Technology and Health Solutions (MS PATHS). Front. Neurol..

[35] Garbarino S., Tur C., Lorenzi M., Pardini M., Piana M., Uccelli A., Arnold D.L., Cree B.A.C., Sormani M.P., Bovis F. (2024). A data-driven model of disability progression in progressive multiple sclerosis. Brain Commun..

[36] International Multiple Sclerosis Genetics Consortium (2019). Multiple sclerosis genomic map implicates peripheral immune cells and microglia in susceptibility. Science.

[37] Andlauer T.F.M., Buck D., Antony G., Bayas A., Bechmann L., Berthele A., Chan A., Gasperi C., Gold R., Graetz C. (2016). Novel multiple sclerosis susceptibility loci implicated in epigenetic regulation. Sci. Adv..

[38] Comabella M., Montalban X. (2014). Body fluid biomarkers in multiple sclerosis. Lancet Neurol..

[39] Uchida Y., Nishimaki K., Soldan A., Pettigrew C., Ho S.G., Moghekar A., Wang M.C., Miller M.I., Albert M., Oishi K. (2025). Change points for dynamic biomarkers in the Alzheimer’s disease pathological cascade: A 30-year cohort study. Alzheimers Dement..

